# Atypical Presentation of Panhypopituitarism

**DOI:** 10.7759/cureus.9102

**Published:** 2020-07-09

**Authors:** Harpreet K Rai, Geevarghese John, Maria Anton

**Affiliations:** 1 Internal Medicine, Northwell Health Long Island Jewish Forest Hills Hospital, Forest Hills, USA; 2 Endocrinology, Diabetes and Metabolism, Northwell Health Long Island Jewish Forest Hills Hospital, Forest Hills, USA

**Keywords:** panhypopituitarism, hypopituitarism, syncope, macroadenoma, sellar mass, secondary adrenal insufficiency, acth, anterior pituitary hormone, deficiency, endocrinology

## Abstract

Hypopituitarism is a rare disorder. Hypopituitarism can present as a deficiency of individual anterior pituitary hormones (e.g., adrenocorticotropic hormone, thyroid-stimulating hormone, luteinizing hormone, follicle-stimulating hormone, prolactin, growth hormone) or posterior pituitary hormones (e.g., oxytocin, vasopressin) or as the deficiency of all these pituitary hormones, also known as panhypopituitarism. Here, we discuss a 59-year-old man who presented with two episodes of unwitnessed syncope after an episode of vomiting. On admission, the patient was hypotensive to 88/54 mmHg, afebrile, and with a leukocyte count of 21.43 K/µL (reference range: 3.80 to 10.50 K/µL). CT scan of the head revealed a hyperdensity in the left intracranial internal carotid artery just proximal to the bifurcation, suggesting an artifact or presence of an embolus. Additional findings included a sellar mass with calcifications and suprasellar extensions. The patient was admitted for further workup of syncope. Other differential diagnoses included sepsis, stroke, cardiac arrhythmias, and pulmonary embolism. Sepsis, stroke, and cardiac workup were negative for significant findings. The patient remained persistently hypotensive despite aggressive intravenous hydration, raising suspicion for an underlying endocrine disorder. MRI of the brain was negative for stroke but again was significant for a sellar mass. Additional workup showed a deficiency of all the anterior pituitary hormones likely secondary to mass effect. The patient was diagnosed with panhypopituitarism due to pituitary macroadenoma.

## Introduction

There are fewer than 200,000 patients with hypopituitarism in the United States [1]. The global incidence is estimated to be 4.2 cases per 100,000 patients per year [1]. A study from northwestern Spain conducted by Regal et al. reported a prevalence of 45.5 cases per 100,000 population [2]. Damage to the anterior pituitary can be mild or severe, can present precipitously or insidiously, and can affect the secretion of one or more of its hormones. As a result, hypopituitarism has a wide range of clinical presentations. Pituitary apoplexy, for example, presents with the rapid development of symptoms causing sudden impairment of adrenocorticotropic hormone (ACTH) secretion and, consequently, the sudden onset of cortisol deficiency symptoms. On the other hand, radiation damage to the pituitary takes time to present. Clinical presentation can also vary, depending on the severity of the hormonal deficiency. The causes of hypopituitarism include pituitary tumor (61%), nonpituitary tumor (9%), or a nontumor cause (30%) [2]. Hypopituitarism can result from diseases of the pituitary gland or diseases of the hypothalamus. The diseases affecting the pituitary gland secretions include mass lesions, surgery, and radiation to treat mass lesions. Hereditary hemochromatosis, hypophysitis (including lymphocytic, granulomatous, plasmacytic, and xanthomatous), pituitary infarction (Sheehan syndrome), pituitary apoplexy, and pituitary infection are among the rare causes of this condition. We describe a patient who presented following a syncopal episode with a wide range of differential diagnoses based on initial laboratory results, clinical course, and workup, ultimately leading to the diagnosis of panhypopituitarism.

## Case presentation

A 59-year-old man with past medical history of hyperlipidemia presented to the ED following two episodes of unwitnessed syncope. The patient reported a sudden onset of nausea and vomiting prior to these episodes. He described a room-spinning sensation when he awoke associated with ringing in the ear. He regained consciousness within one to two minutes. While attempting to stand, he again lost consciousness. In the ED, the patient denied any symptoms.

In the ED, his physical examination was significant for symptomatic orthostatic hypotension. Vitals included blood pressure of 88/54 mmHg and a heart rate of 85 beats/minute. The patient was afebrile. The patient's initial laboratory workup results are presented in Table 1. His electrocardiogram showed normal sinus rhythm. His chest X-ray findings were unremarkable. A CT scan of the patient's head showed an apparent hyperdensity in the left intracranial internal carotid artery just proximal to the bifurcation, representing an artifact or the presence of an embolus. It also showed an apparent sellar mass with calcifications and suprasellar extension. A CT scan of the abdomen showed cholelithiasis and was negative for any acute pathology.

**Table 1:** Initial laboratory test results

Analyte	Results	Reference range
Leukocyte	21.43 K/µL	3.80-10.50 K/µL
Hemoglobin	11.3 g/dL	11.5-15.5 g/dL
Blood urea nitrogen, Serum	31 mg/dL	7-18 mg/dL
Creatinine, Serum	1.31 mg/dL	0.50-1.30 mg/dL
Sodium, Serum	135 mmol/L	135-145 mmol/L
Potassium, Serum	3.9 mmol/L	3.5-5.3 mmol/L
Bicarbonate, Serum	27 mmol/L	22-31 mmol/L
Troponin I, Serum	<0.015 ng/mL	0.000-0.045 ng/mL
Urine analysis	Negative	

The patient was admitted for further evaluation of syncope. Sepsis was considered given his leukocytosis and hypotension. A CT scan of the head showing a questionable embolus was followed by a neurological evaluation to test for a possible stroke. The patient was admitted to a telemetry unit to rule out syncope due to cardiac arrhythmias. Intravenous fluids were started to treat his hypotension.

On the patient's second day of admission, the intensive care unit team was consulted for persistent hypotension. Intravenous hydration was recommended along with a follow-up workup for sepsis and pulmonary embolism. A morning cortisol level was also ordered to rule out adrenal insufficiency. The patient was not started on antibiotics at that time as he remained afebrile, the leukocyte count normalized, and the blood culture showed no bacterial growth. A CT angiogram of his chest was significant for a small filling defect in a right upper lobe segmental pulmonary artery, compatible with pulmonary embolism. The patient was not started on therapeutic anticoagulation as a follow-up ventilation-perfusion scan showed low probability for pulmonary embolism.

**Figure 1:**
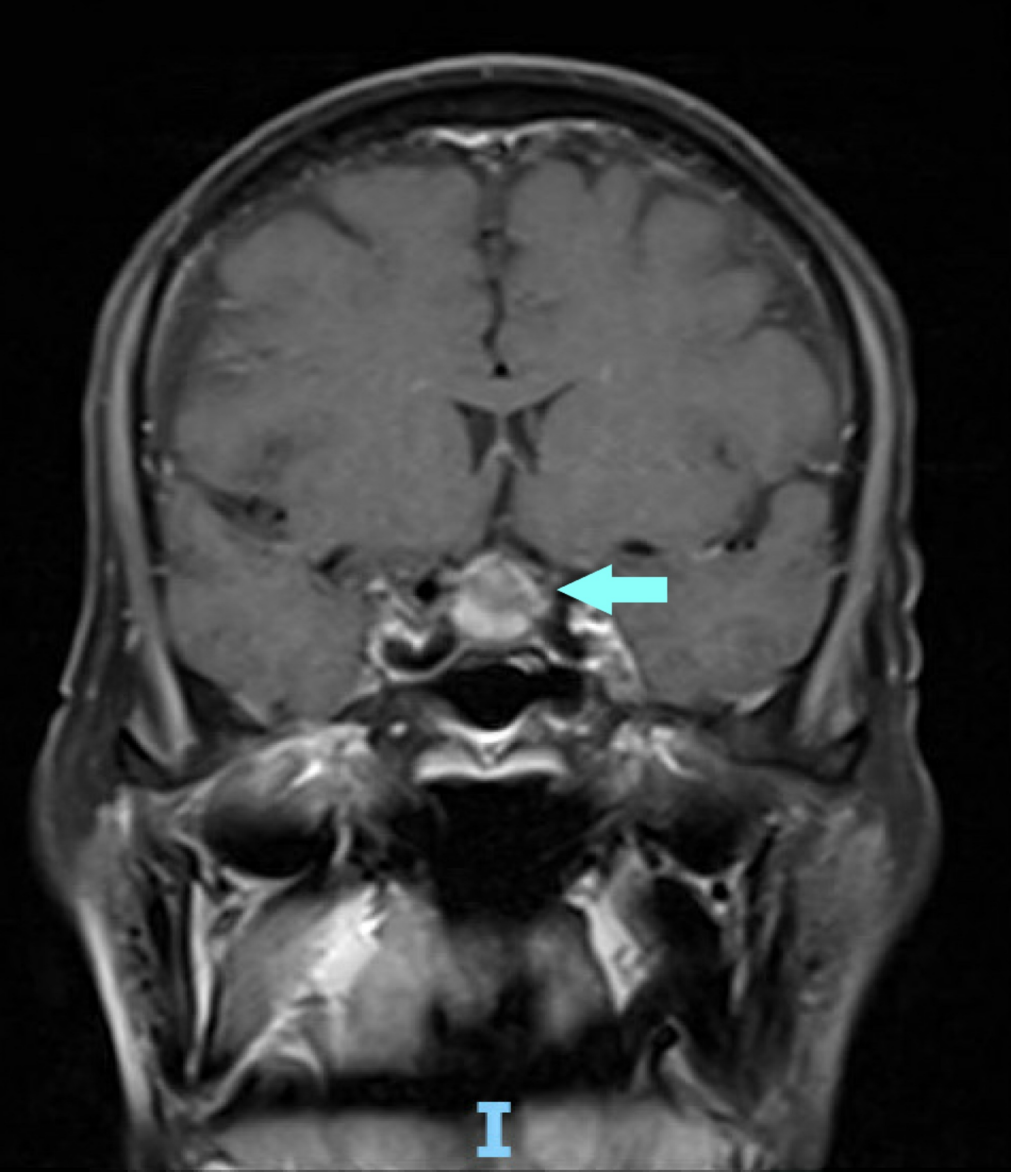
Magnetic resonance imaging showing pituitary macroadenoma in coronal view

**Figure 2:**
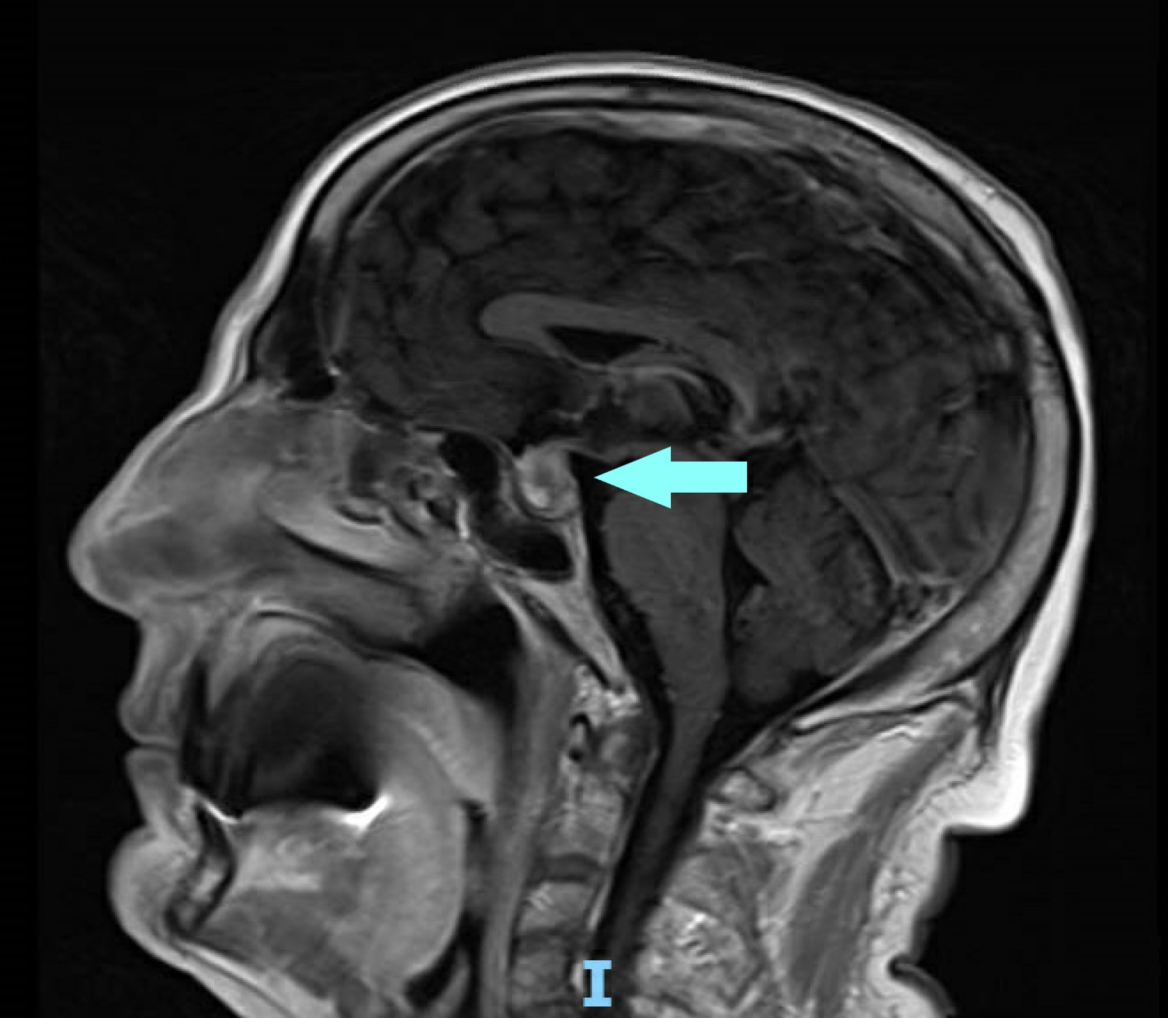
Magnetic resonance imaging showing pituitary macroadenoma in sagittal view

The patient underwent a cardiology evaluation for syncope and suspected underlying arrhythmia. Telemetry monitoring was negative for significant cardiac arrhythmias. Echocardiography showed an ejection fraction of 55%, normal diastolic function, and normal valves. The results of his neurological exam were benign, as the patient had no focal weakness and reflexes were normal. MRI of the brain with contrast was negative for a stroke but was significant for an enhancing mass filling and expanding the sella turcica, measuring 1.5 cm caudally, 1.2 cm anteroposteriorly, and 1.5 cm transversely. It encroached on and elevated the optic chiasm (Figures 1, 2).

On the patient's third day after admission, his morning cortisol level was inappropriately low (1.6 µg/dL; reference range: 6.0 to 18.4 µg/dL). The endocrinology team was consulted over concerns for secondary adrenal insufficiency due to the sellar mass. A cosyntropin stimulation test showed maximum cortisol elevation to 10.1 µg/dl. Table 2 present the patient's hormone level results. He was diagnosed with central hypothyroidism, then panhypopituitarism due to the pituitary macroadenoma. The patient was started on hydrocortisone, 10-mg tablet in the morning and 5-mg tablet in the evening, along with levothyroxine, 50-mcg tablet once daily.

**Table 2:** Patient hormone levels, day 3

Hormone	Result	Reference Range
Cortisol AM, Serum	1.6 µg/dL	6.0-18.4 µg/dL
Thyroid-stimulating hormone, Serum	1.56 µU/mL	0.34-4.82 µU/mL
Total thyroxine, Serum	4.4 µg/dL	4.6-12.0 µg/dL
Free thyroxine, Serum	0.4 ng/dL	0.9-1.8 ng/dL
Triiodothyronine, Total Serum	43.0 ng/dL	80-200 ng/dL
Prolactin, Serum	13.6 ng/mL	4.1-18.4 ng/mL
Follicle-stimulating hormone, Serum	0.7 IU/L	1.5-12.4 IU/L
Luteinizing hormone, Serum	<0.3 IU/L	1.24-7.80 IU/L
Testosterone, Total Serum	<2.5 ng/dL	193.0-740.0 ng/dL
Insulin-like growth factor 1	51 ng/mL	55-103 ng/mL

The patient was discharged with recommendations for outpatient follow-up with the endocrinology team for continued monitoring of his pituitary hormone level deficiencies and sellar mass. Outpatient ophthalmology follow-up was also recommended for visual field assessment, as the mass was causing elevation of the optic chiasm. Finally, his care team recommended outpatient neurosurgery follow-up evaluation for possible surgical resection of the mass. The outpatient follow-up assessments were ultimately delayed due to the novel coronavirus disease (COVID-19) pandemic.

## Discussion

Syncope is a common presenting symptom in the ER, accounting for 3% to 5% of ER visits and a hospitalization rate in approximately 40% of cases. The average length of stay for evaluation of syncope is 5.5 days [3]. After reviewing the history, physical examination findings, and electrocardiogram, ER physicians can determine the underlying diagnosis in approximately 50% of cases [4]. Patients admitted to the hospital for further workup of syncope usually undergo an extensive cardiac and neurological assessment. According to a study performed on 433 patients with syncope, an etiology could not be identified in 41% of patients [5]. 

The patient presented above was diagnosed with a sellar mass leading to hyposecretion of all the pituitary hormones. His symptoms were mainly related to ACTH deficiency (secondary adrenal insufficiency). Symptoms of ACTH deficiency are exclusively related to cortisol deficiency. Cortisol is necessary for peripheral vascular tone. In its most severe form, cortisol deficiency leads to death due to vascular collapse. Milder symptoms of the same phenomenon include postural hypotension and tachycardia. An ACTH deficiency does not affect aldosterone secretion (which is present in primary adrenal insufficiency); therefore, it does not cause salt wasting, volume contraction, or hyperkalemia. Hyponatremia can be present because cortisol deficiency can cause inappropriate secretion of antidiuretic hormone (vasopressin). Our patient presented with normal electrolytes as mentioned in Table 1, that is, he did not have signs of salt wasting or volume contraction. ACTH deficiency does not cause hyperpigmentation, which is a symptom of primary adrenal insufficiency due to a reflex increase in ACTH.

The treatment of hypopituitarism is focused on the supplementation of the individual deficient hormones to restore the body's natural physiology. This provides relief of symptoms with minimal side effects. The patient was started on hydrocortisone and levothyroxine supplementation. Outpatient endocrinology follow-up was recommended for continued hormone monitoring and testosterone replacement therapy. Transsphenoidal surgery is the mainstay of treatment for most sellar masses and would be a potential therapy for this patient. However, due to the COVID-19 pandemic, the outpatient follow-up has been delayed.

## Conclusions

Syncope is a common presenting symptom in the ER. Postural hypotension and syncope are the most common presentations of secondary adrenal insufficiency and panhypopituitarism. Evaluation of syncope rarely includes a thorough endocrine workup. In this case, endocrinology workup was started after neurological, cardiac, and septic evaluations were insignificant. Clinicians should be mindful of considering endocrine causes as a differential for syncope at the time of presentation.
